# Saline Infusion in Puerperal Eclampsia

**Published:** 1901-05-18

**Authors:** 


### SALINE INFUSION IN PUERPERAL ECLAMPSIA.

At the last meeting of the Obstetrical Society the treatment of puerperal eclampsia by saline infusion was discussed. Varied opinions were expressed as to its utility. Probably the truth is that what is called puerperal eclampsia is not always the same disease; sometimes being an expression of a condition definitely connected with pregnancy, while in others it is the result of an accidental complication of the pregnancy with co-existing disease of the kidneys. Dr. Hey Groves had advocated repeated saline transfusion when there were profound coma, cyanosis and anuria, with chloroform during delivery. In summing up the matter the President (Dr. Peter Horrocks) said that he was not convinced that in saline transfusion they had a remedy for puerperal eclampsia. If used he thought it was perhaps better to inject it not into the veins but into the cellular tissue or rectum. It must be remembered that in these cases there had been no loss of blood, and hence there was no underfilling of the vascular system as there was in cases of profound hæmorrhage. The blood pressure was generally raised, and it might therefore not be without danger to add rapidly several pints of fluid. The vessels, however, would not absorb any dangerous amount from the cellular tissue, the process being one of osmosis. He could not help thinking, he said, that if there were any truth in the theory that puerperal eclampsia was due to a toxin circulating in the blood, and any truth in the theory that diluting the blood would cure or mitigate the fits, then blood should be taken from one median basilic vein while saline fluid was injected into the vein on the opposite side. This seems reasonable.

